# Pulmonary gas exchange in hereditary hemorrhagic telangiectasia patients with liver arteriovenous malformations

**DOI:** 10.1186/s12931-019-1106-y

**Published:** 2019-07-04

**Authors:** Carole de Picciotto, Mostafa El Hajjam, Carma Karam, Thierry Chinet, Marcel Bonay

**Affiliations:** 1 Service de Physiologie-Explorations Fonctionnelles Hôpital Ambroise Paré Assistance Publique-Hôpitaux de Paris (AP-HP) Boulogne, France et Université de Versailles Saint Quentin (UVSQ), 92104 Boulogne cedex, Boulogne, France; 2 HHT center Hôpital Ambroise Paré Assistance Publique-Hôpitaux de Paris (AP-HP) Boulogne, France et Université de Versailles Saint Quentin (UVSQ), Boulogne, France; 3 Service de Radiologie Hôpital Ambroise Paré Assistance Publique-Hôpitaux de Paris (AP-HP) Boulogne, France et Université de Versailles Saint Quentin (UVSQ), Boulogne, France; 4 Service de Cardiologie Hôpital Ambroise Paré Assistance Publique-Hôpitaux de Paris (AP-HP) Boulogne, France et Université de Versailles Saint Quentin (UVSQ), Boulogne, France; 5 Service de Pneumologie Hôpital Ambroise Paré Assistance Publique-Hôpitaux de Paris (AP-HP) Boulogne, France et Université de Versailles Saint Quentin (UVSQ), Boulogne, France

**Keywords:** Pulmonary diffusing capacity, Arteriovenous malformations, Hereditary hemorrhagic telangiectasia, Computed tomography scan of the liver and chest, Echocardiography

## Abstract

**Background:**

The severity of Hereditary Hemorrhagic Telangiectasia (HHT) disease is generally related to vascular visceral involvement represented by arteriovenous malformations (AVMs). Pulmonary function tests (PFTs) remain normal in HHT patients without Pulmonary AVMs (PAVMs) and respiratory comorbidity. The aim of our study was to compare the diffusing capacity of the lung for carbon monoxide (D_LCO_) and nitric oxide (D_LNO_) and its 2 components: the pulmonary capillary blood volume (Vc) and the alveolar-capillary membrane conductance (Dm), in HHT patients with PAVMs, PAVMs and liver AVMs (LAVMs), LAVMs without PAVM, no PAVM and LAVM, and controls.

**Methods:**

Sixty one consecutive adult patients (HHT without PAVM and LAVM: *n* = 7; HHT with PAVMs: *n* = 8; HHT with PAVMs and LAVMs: *n* = 25; HHT with LAVMs: *n* = 21) and controls matched for age and sex ratio without respiratory, heart and liver pathology (*n* = 15) were non-invasively evaluated using PFTs, combined D_LCO_/D_LNO_, arterial blood gas at rest, contrast echocardiography and enhanced computed tomography scan of the liver and chest the day of pulmonary function testing.

**Results:**

We found that patients with LAVMs but without PAVMs exhibited increased Vc/Dm ratio. Interestingly, HHT patients with hepatic artery enlargement showed higher Vc/Dm ratio than HHT patients with normal hepatic artery diameter.

**Conclusion:**

Vc/Dm ratio may have practical impact in HHT patients’ management to detect precociously the occurrence of LVAMs. However, further studies are needed to assess the accuracy and potential prognostic value of pulmonary gas exchange measurements in HHT patients with LVAMs.

## Introduction

The severity of Hereditary Hemorrhagic Telangiectasia (HHT) or Rendu-Osler-Weber disease is generally related to vascular visceral involvement represented by arteriovenous malformations (AVMs). Mutations in the endoglin (ENG) gene, the activin A receptor type II-like 1 (ACVRL1/ALK1) gene, and infrequently, the SMAD4 gene are responsible for HHT disease [1]. Pulmonary AVMs (PAVMs) induce right-to-left shunting, which is routinely assessed by contrast echocardiography and arterial blood gas measurements. Pulmonary functions test (PFTs), completed concomitantly with arterial blood gas measurements, remain normal in HHT patients without PAVMs and respiratory comorbidity [2]. Nevertheless, few studies have evaluated pulmonary gas exchange in HHT patients. The combined diffusing capacity of the lung for carbon monoxide (D_LCO_) and nitric oxide (D_LNO_) is a simple test that allows the determination of 2 components of pulmonary gas exchange: the pulmonary capillary blood volume (Vc) and the alveolar-capillary membrane conductance (Dm) [3].

The aim of our study was to compare diffusing capacity of the lung for carbon monoxide (D_LCO_) and nitric oxide (D_LNO_) and its 2 components Dm and Vc, in HHT patients with PAVMs, PAVMs and liver AVMs (LAVMs), LAVMs without PAVM, no PAVM and LAVM, and controls.

## Methods

To be included in the study, patients had to fulfill the following criteria: (1) diagnosis of HHT ascertained by identification of an ENG, ACVRL1/ALK1, or SMAD4 mutation and/or by the presence of at least three Curaçao criteria (nose bleeds, mucocutaneous telangiectasia, visceral arteriovenous malformations, and family history) and (2) age > 18 years. Patients were not included if they were suffering from respiratory or cardiac conditions unrelated to HHT. Sixty one consecutive adult patients with forced expiratory volume in one second/vital capacity ratio > 70% (HHT without PAVM and LAVM: *n* = 7; HHT with PAVMs: *n* = 8; HHT with PAVMs and LAVMs: *n* = 25; HHT with LAVMs: *n* = 21) and controls matched for age and sex ratio without respiratory, heart and liver pathology (*n* = 15) were non-invasively evaluated, using PFTs, combined D_LCO_/D_LNO_, arterial blood gas at rest, contrast echocardiography and enhanced computed tomography scan of the liver and chest the day of pulmonary function testing (Table 1).

**Table 1:** Characteristics of the study populations

	Controls	HHT	PAVMs	P + LAVMs	LAVMs
n	15	7	8	25	21
Age, yr. ^a^	46 (23;53)	27 (18;59)	40 (35;51.75)	36 (29;47)	43.7 (32.5;55.5)
Female/Male ratio ^a^	10/5	2/5	4/4	16/9	17/4
LVEF,% ^a^	nd	69 (61;73)	63 (61.5;71.75)	70 (67;76)	71 (68.5;75.5)
(A-a) O_2_^a^ (kPa)	nd	1.25 (0;2.92)	2 (1.5;2.85)	2 (0.5;3.7)	1.15 (0.5;1.75)
Hb, g/dL ^a^	nd	14.9 (14.5;15.1)	14.55 (13.8;15.2)	14.2 (13.5;15)	14 (12.1;14.7)
TLC, % ^a^	93.8 (90;100)	99 (90;102)	104.5 (99.25;113)	104 (94.5;111.5)	101 (91;117.5)
DLCO,% ^a^	88.5 (85;92.8)	85 (80;97)	104 (91.75;110)	81 (74.5;86.5)	91 (79.5;98.5)
DLCO/VA,% ^a^	95.2 (90.6;112.9)	98 (92.5;102.5)	100 (78;111)	88 (81;95)	94 (86.25;104)
D_LNO_/D_LCO_	4.1 (3.8;4.3)	4.5 (4.3;4.7)#	4.3 (4.2;4.6)	4.5 (4.25;4.6)#	4.3 (3.95;4.5)
Vc, % ^a^	85 (76.7;91.9)	85 (79;93)	103.5 (94.25;110.8)	78 (73;89)	94 (79;97)
Dm, % ^a^	125.5 (120.7;139.2)	113 (102;119)	135.5 (129.3;147.3)	107 (98.5;116)	117 (100;125.5)
Vc/Dm ratio	1.09 (1.01;1.2)	1.15 (1.13;1.25)	1.21 (1.12;1.26)	1.16 (1.11;1.42)	1.35 (1.18;1.62)^b^

Values given as median (25%;75% quartiles), ^a^ no difference between groups; ^b^: *p* < 0.05 vs other groups; # *p* = 0.01 vs controls; *HHT* Hereditary Hemorrhagic Telangiectasia patients without arteriovenous malformations, *PAVMs* pulmonary arteriovenous malformations, *P + LAVMs* Pulmonary+Liver arteriovenous malformations, *LAVMs* Liver arteriovenous malformations. *LVEF* left ventricular ejection fraction, *(A-a) O*_*2*_ alveolar-arterial O_2_ difference at rest, *Hb* hemoglobin level g/dL, *TLC, %* total lung capacity, % predicted, *D*_*LCO*_ diffusing capacity of the lung for carbon monoxide, *D*_*LCO*_*/V*_*A*_ transfer coefficient for carbon monoxide, *Vc* pulmonary capillary blood volume, *Dm* alveolar-capillary membrane conductance, *D*_*LNO*_*/D*_*LCO*_ diffusing capacity of the lung for nitric oxide/D_LCO_ ratio

Lung volumes were measured with a PFT-Masterscreen apparatus (Jaeger, CareFusion, Hoechberg, Germany). PFTs included measurements of functional residual capacity (FRC) by the helium dilution method and calculation of total lung capacity (TLC), measurements of vital capacity (VC) and forced expiratory volume in 1 s (FEV_1_). Arterial blood sampling was made with patients in the sitting position. Blood sample was analyzed for pO_2_, pCO_2_, pH and hemoglobin concentration (Rapidlab 1245, Siemens Healthcare Diagnostics Inc., Tarrytown, NY, USA).

Simultaneous measurements of D_LNO_, D_LCO_ and V_A_ were performed using the single-breath method (MasterScreen™ PFT Pro, Jaeger, CareFusion, Hoechberg, Germany). Hemoglobin level (Hb) was measured in the patients and D_LCO_ was corrected to the standard Hb according to international guidelines [3, 4]. Dm was expressed as D_LNO_ divided by 1.97 [5], and Vc was derived as previously described [6].

Systolic pulmonary artery pressure (sPAP) was noninvasively assessed by Doppler echocardiography (Siemens/Sequoia Acuson C512 system (Acuson, Mountain View, CA, USA) or Vivid E9 (GE Medical Systems, Horten, Norway) from tricuspid regurgitation using the modified Bernoulli equation [7]. Left ventricular ejection fraction was calculated by the method of Teichholz.

Computed tomography scan of the liver and chest were performed by using a 16–detector row helical CT scanner (Mx 8000 IDT; Philips Healthcare, Best, the Netherlands). Pulmonary involvement (identification of pulmonary AVMs) and hepatic involvement (dilatation of the hepatic artery > 6 mm, telangiectasia, or vascular confluent masses or intrahepatic vascular shunts) were recorded. The diameter of the common hepatic artery was measured 1 cm before the origin of the gastroduodenal artery [8].

Institutional review board approval was obtained for this study (CEPRO 2014–047). Statistical analyses were performed using GraphPad Prism 5.01 for Windows (GraphPad Software, San Diego California). Between-group differences were first assessed using analysis of variance with Bonferroni correction for multiple comparisons followed by post-hoc Mann-Whitney test. Results were reported as the median and 25 and 75% quartiles. A *p* value < 0.05 was considered statistically significant.

## Results

**Fig. 1:**
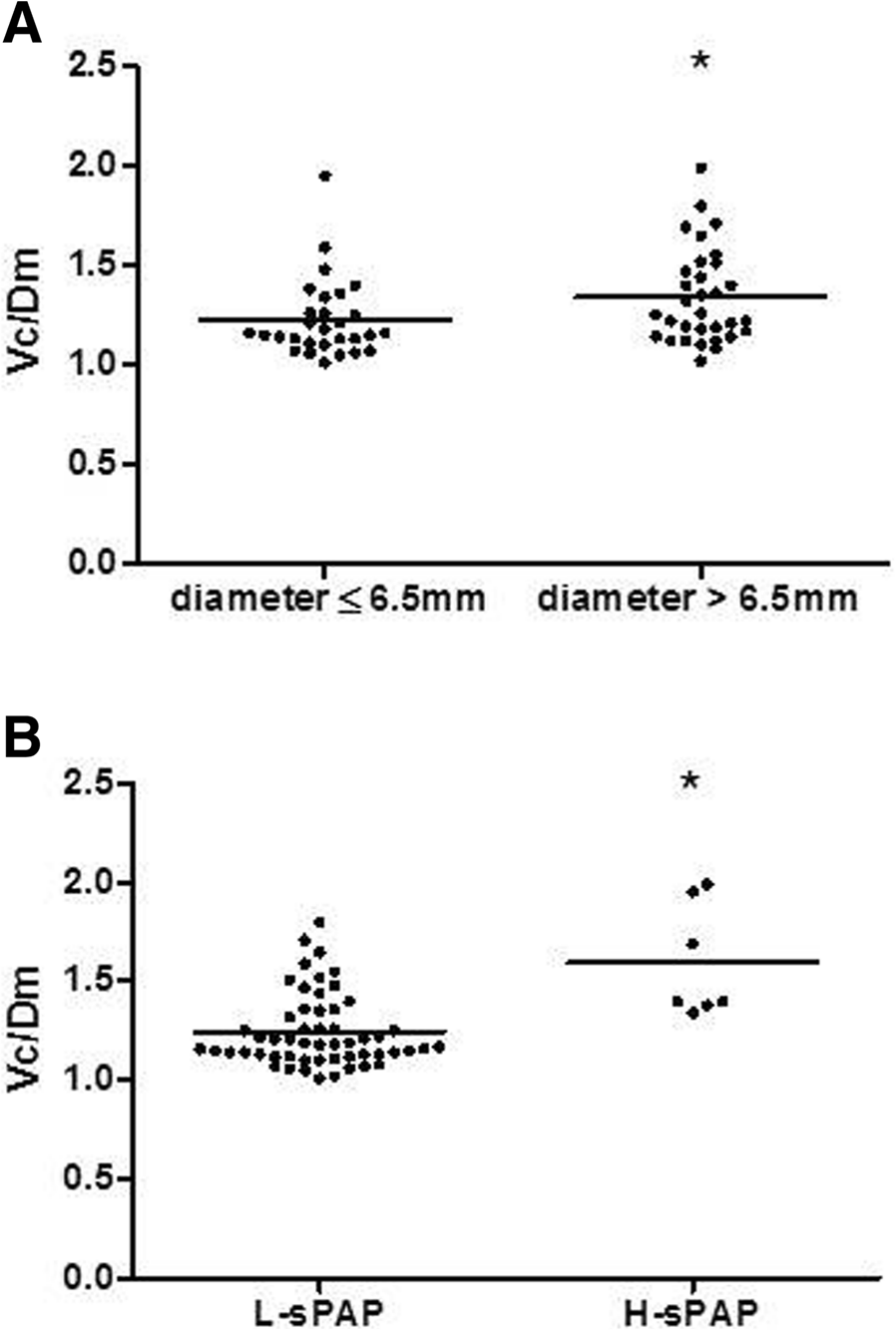
**a** Pulmonary capillary blood volume/alveolo-capillary membrane conductance ratio (Vc/Dm) was increased in Hereditary Hemorrhagic Telangiectasia (HHT) patients with hepatic artery diameter > 6.5 mm vs HHT patients with hepatic artery diameter ≤ 6.5 mm, * *p* = 0.03; **b** Pulmonary capillary blood volume/alveolo-capillary membrane conductance ratio (Vc/Dm) was increased in Hereditary Hemorrhagic Telangiectasia (HHT) patients with high systolic pulmonary artery pressure (H-sPAP ≥37 mmHg) vs HHT patients with low sPAP (L-sPAP < 37 mmHg) and controls, * *p* < 0.01

Total lung capacity, alveolar-arterial O_2_ difference at rest, hemoglobin level, D_LCO_, transfer coefficient for carbon monoxide (D_LCO_/V_A_), Vc and Dm (% predicted) did not differ between groups (Table 1). Assuming that D_LNO_ represents the alveolar membrane component of gas transfer, and that D_LCO_ depends on Dm, Vc, hemoglobin concentration and pulmonary capillary oxygen tension, the D_LNO_/D_LCO_ ratio might differ between patients with alveolocapillary membrane modifications and patients with microvascular disease. D_LNO_/D_LCO_ ratio was increased in HHT patients (*p* = 0.01) and in P + LAVM patients (*p* = 0.01) versus controls but did not differ between patients groups (Table 1). Vc scaled by Dm (Vc/Dm ratio), which is used to assess the functional efficiency of the alveolar-capillary membrane, was increased (*p* < 0.05 for all) in LAVMs as compared to other groups (Table 1). When a cut-off value of 6.5 mm diameter was used to establish common hepatic artery enlargement [8], HHT patients with hepatic artery enlargement (diameter > 6.5 mm) showed higher Vc/Dm ratio than HHT patients with hepatic artery diameter ≤ 6.5 mm (1.25 (1.15;1.50), *n* = 32 vs 1.16 (1.10;1.30), *n* = 29; *p* = 0.03) (Fig. 1a). Vc/Dm ratio was decreased in HHT patients with low systolic pulmonary artery pressure < 37 mmHg, a cut-off value associated with no indirect echographic signs of pulmonary hypertension [9], as compared to HHT patients with high systolic pulmonary artery pressure ≥ 37 mmHg (1.18 (1.12;1.35), *n* = 54 vs 1.40 (1.38;1.95), *n* = 7; *p* < 0.01) (Fig. 1b).

## Discussion

Our results suggest that pulmonary gas exchanges are modified in HHT patients with LAVMs. More specifically, we found that patients with LAVMs but without PAVMs exhibited increased Vc/Dm ratio.

A small decrease in the transfer coefficient for carbon monoxide (D_LCO_/V_A_) has been reported in HHT patients in some studies [10, 11]. However, the effect of HHT on 2 components of pulmonary gas exchange Vc and Dm was not determined and comparisons between patients with or without arteriovenous malformations and controls were not done. In our study, we found no difference in the diffusing capacity of the lung for carbon monoxide in HHT patients with or without arteriovenous malformations and in control patients.

The mechanism involved in the increase in the Vc/Dm ratio in HHT patients with LAVMs has not been clearly defined. Left ventricular ejection fraction obtained by echocardiography was normal in our study population, but cardiac output was not systematically measured. Interestingly, in a recent work, low Dm/Vc ratio was associated with failure to achieve the high cardiac output of hyperdynamic state of obesity [12]. The possible increased pulmonary blood flow secondary to the increased cardiac output state associated with LAVMs in HHT patients might contribute to the high Vc/Dm ratio. The higher Vc/Dm ratio found in HHT patients with hepatic artery enlargement fits well with this hypothesis as a cut-off value of 6.5 mm diameter was used to establish common hepatic artery enlargement and hepatic involvement in HHT [8].

In our study, Vc/Dm ratio was increased in HHT patients with high systolic pulmonary artery pressure. Increased pulmonary capillary blood volume secondary to increased pulmonary pressure observed in heart failure is unlikely because left ventricular ejection fraction assessed by echocardiography was normal in our study population. Interestingly, one study reported a significant correlation between systolic pulmonary artery pressure and Vc/Dm ratio in patients with chronic infiltrative lung disease and controls [13]. Pulmonary hypertension may develop in HHT patients as a consequence of high output cardiac failure associated with LVAMs [14]. Although right heart catheterization measurements taken as a gold standard were not completed in our study population, transthoracic echocardiography Doppler allows for accurate measurements of the pulmonary circulation [9]. Unfortunately, cardiac output was not systematically measured to detect high output cardiac failure in our patients.

The main result of this work is that patients with isolated liver arteriovenous malformations (LAVMs) display an increased pulmonary capillary blood volume over alveolar-capillary membrane conductance ratio (Vc/Dm), which may be due to higher cardiac output. However, some limitations should be highlighted. First, right heart catheterization was not done and cardiac output was not systematically measured. Further studies including these measurements are needed to confirm that high pulmonary blood flow, secondary to increased cardiac output, might contribute to the increase in the Vc/Dm ratio in LAVMs patients. Secondly, the Vc/Dm ratio was not increased in patients with liver and pulmonary arteriovenous malformations (P + LAVMs). Different haemodynamic profiles have been described in LAVMs and P + LAVMs patients [15]. However, the mechanisms of these cardiovascular modifications and their consequences on pulmonary circulation and gas exchange are not fully understood.

## Conclusions

LVAMs may alter pulmonary gas exchange in HHT patients as reflected by the increase in the Vc/Dm ratio, which is possibly induced by the increase in the cardiac output. This pulmonary gas exchange alteration may have practical impact in HHT patients’ management. Indeed, as combined D_LCO_/D_LNO_ assessment is simple and inexpensive, this test may be used for fine follow-up of HHT patients to detect precociously the occurrence of LVAMs. However, further studies are needed to assess the accuracy and potential prognostic value of pulmonary gas exchange measurements in HHT patients with LVAMs as compared with right heart catheterization measurements and computed tomography scan of the liver and chest.

## Data Availability

The authors declare that the data supporting the findings of this study are available within the article.

## Abbreviations

AVMs: Arteriovenous malformations
D_LCO_: Diffusing capacity of the lung for carbon monoxide
D_LCO_/V_A_: Transfer coefficient for carbon monoxide
D_LNO_: Diffusing capacity of the lung for nitric oxide
Dm: Alveolo-capillary membrane conductance
HHT: Hereditary Hemorrhagic Telangiectasia
LAVMs: Liver AVMs
PAVMs: Pulmonary AVMs
PFTs: Pulmonary function tests
sPAP: Systolic pulmonary artery pressure
Vc: Pulmonary capillary blood volume
